# Tyrosine phosphorylation of band 3 impairs the storage quality of suspended red blood cells in the Tibetan high-altitude polycythemia population

**DOI:** 10.1186/s12967-023-04428-5

**Published:** 2023-09-28

**Authors:** Xiaodong Wu, Zhijuan Liu, Doudou Hao, Qin Zhao, Wanjing Li, Maodi Xie, Xia Feng, Xia Liao, Siyuan Chen, Siyu Wang, Chaohua Zhou, Wenchun Long, Yajun Zhong, Shen Li, Ye Cao, Hong Wang, Aiping Wang, Yuehong Xu, Min Huang, Jiaxin Liu, Rui Zhong, Yunhong Wu, Zeng He

**Affiliations:** 1 Department of Critical Care Medicine, Hospital of Chengdu Office of People’s Government of Tibetan Autonomous Region, Chengdu, 610041 People’s Republic of China; 2 Department of Blood Transfusion, People’s Hospital of Tibet Autonomous Region, Lhasa, 851400 Tibet People’s Republic of China; 3 Department of Biobank, Hospital of Chengdu Office of People’s Government of Tibetan Autonomous Region, Ximianqiao Rd #20, Wuhou District, Chengdu, 610041 People’s Republic of China; 4 Center of Biomedical Engineering, Institute of Blood Transfusion, Chinese Academy of Medical Sciences and Peking Union Medical College, Huacai Rd #26, Chenghua District, Chengdu, 610052 People’s Republic of China; 5 Laboratory of Mitochondria and Metabolism, Department of Anesthesiology, National Clinical Research Center for Geriatrics, West China Hospital of Sichuan University, Chengdu, 610041 China; 6 Department of Endocrinology and Metabolism, Hospital of Chengdu Office of People’s Government of Tibetan Autonomous Region, Ximianqiao Rd #20, Wuhou District, Chengdu, 610041 People’s Republic of China; 7 Department of Blood Transfusion, Hospital of Chengdu Office of People’s Government of Tibetan Autonomous Region, Chengdu, 610041 People’s Republic of China

## Abstract

Due to environmental hypoxia on the Tibetan Plateau, local residents often exhibit a compensative increase in hemoglobin concentration to maintain the body’s oxygen supply. However, increases in hemoglobin and hematocrit (Hct) pose a serious challenge to the quality of stored suspended red blood cells (SRBCs) prepared from the blood of high-hemoglobin populations, especially populations at high altitude with polycythemia in Tibet. To explore the difference in storage quality of SRBCs prepared from plateau residents with a high hemoglobin concentration, blood donors were recruited from Tibet (> 3600 m) and Chengdu (≈ 500 m) and divided into a high-altitude control (HAC) group, high-altitude polycythemia (HAPC) group and lowland control (LLC) group according to their hemoglobin concentration and altitude of residence. The extracellular acidification rate (ECAR), pyruvate kinase (PK) activity and band 3 tyrosine phosphorylation were analyzed on the day of blood collection. Then, whole-blood samples were processed into SRBCs, and storage quality parameters were analyzed aseptically on days 1, 14, 21 and 35 of storage. Overall, we found that tyrosine 21 phosphorylation activated glycolysis by releasing glycolytic enzymes from the cytosolic domain of band 3, thus increasing glucose consumption and lactate accumulation during storage, in the HAPC group. In addition, band 3 tyrosine phosphorylation impaired erythrocyte deformability, accompanied by the highest hemolysis rate in the HAPC group, during storage. We believe that these results will stimulate new ideas to further optimize current additive solutions for the high-hemoglobin population in Tibet and reveal new therapeutic targets for the treatment of HAPC populations.

## Introduction

With the development and application of additive solutions, suspended red blood cells (SRBCs) prepared from whole blood can be stored at 4 °C for up to 35–42 days (depending on the composition of the additive solutions used). The extended storage of blood components is a logistical necessity that permits SRBCs to be available for transfusion with the least delay and has greatly promoted the development of transfusion medicine and the establishment of blood banks [1]. Although in vitro storage at 4 °C greatly restrains the metabolism of RBCs, it is not completely suppressed [1, 2]. Storage of SRBCs in additive solutions results in the progressive accumulation of a series of biochemical, immunological, and biomechanical changes, which are collectively termed “storage lesions” [3]. It is widely accepted that adverse clinical outcomes of RBC transfusion are associated with storage lesions [4–6]. Therefore, restraining storage lesions of RBCs has been a hotspot in the research field of transfusion medicine.

However, the dosages of additive solutions used in the preparation of SRBCs are based on relevant research on lowland populations. Due to environmental hypoxia on the Tibetan Plateau, the hemoglobin concentration of plateau residents often exhibits a compensatory increase to maintain the body’s oxygen supply. This increase in hemoglobin concentration and hematocrit (Hct) would accelerate glucose consumption and generate more lactate during storage, posing a serious challenge to the storage quality of SRBCs prepared from the blood of the Tibetan high-hemoglobin population, especially the high-altitude polycythemia (HAPC) population. HAPC is a clinical syndrome characterized by excessive erythrocytosis (females Hb ≥ 190 g/L; males Hb ≥ 210 g/L) [7], as defined by the International Society for Mountain Medicine (ISMM) at the VI World Congress on Mountain Medicine and High-Altitude Physiology.

In our previous small-scale blood storage experiment, we found significant differences in metabolic parameters between SRBCs prepared from the HAPC population and lowland population [8]. However, due to the restrictions of objective conditions and economic reasons, there were some imperfections in our previous 2015 study. First, volunteers of the HAPC group were recruited and whole-blood was collected in Lhasa, whole-blood samples were transported to Chengdu once a week for subsequent preparation of SRBCs and storage experiments. It means that whole-blood samples of the HAPC group collected at an earlier time had been stored at 4 ℃ for an extra week in Lhasa before SRBCs preparation. Second, the transportation process from Lhasa to Chengdu may also have an impact on the quality of whole-blood samples. Third, the sample numbers of the HAPC and LLC groups were eight, which was insufficient for analysis. Moreover, the underlying mechanism that causes differences in metabolism between the HAPC and LLC groups was unclear.

Therefore, in this work, to exclude the influence of inconsistent time of blood collection and transportation on the storage quality of SRBCs, and clarify the underlying mechanism of more lactate generated in SRBCs prepared from the Tibetan HAPC population, a new recruitment was conducted by our research team in Lhasa for 2 months. First, Tibetan volunteers were transferred to Chengdu, and blood was collected within 30 days of leaving the Tibetan Plateau for subsequent preparation of SRBCs and storage experiments. Second, to investigate the effect of high hemoglobin concentration and Hct on the storage quality of SRBCs, blood donors were recruited from Tibet (> 3600 m) and Chengdu (≈ 500 m) and divided into a high-altitude control (HAC) group, a HAPC group and a lowland control (LLC) group according to hemoglobin concentration and altitude of residence. In brief, volunteers in the HAPC group were recruited from Tibet as an extreme representative of the high-hemoglobin population in Tibet. Volunteers with a normal hemoglobin concentration (120 g/L < males ≤ 185 g/L, 115 g/L < females ≤ 165 g/L) recruited from Tibet and Chengdu were set as the HAC group and LLC group, respectively. In addition, the sample size of each group increased compared to our previous study, however, the sample size was still limited due to problems such as volunteer recruitment.

Moreover, we analyzed differences in glycolytic capacity among the HAC, HAPC and LLC groups by detecting the extracellular acidification rate (ECAR) on the day of blood collection. Then, influencing factors that may affect glycolysis in RBCs, such as the activity of glycolytic enzymes and phosphorylation of band 3, were also analyzed. Band 3, also known as anion exchanger 1 (AE1), is the most abundant protein in the RBC membrane and has diverse biological functions: the cytosolic domain contains binding sites for cytoskeletal proteins, glycolytic enzymes (glyceraldehyde-3-phosphate dehydrogenase, aldolase, phosphofructokinase) and deoxyhemoglobin, and the membrane domain of band 3 mediates anion transport [9, 10]. Furthermore, the phosphorylation of band 3 releases glycolytic enzymes from the membrane to the cytoplasm; thus, the tyrosine phosphorylation of band 3 plays an important role in regulating the metabolic flux of erythrocytes [11–13]. For instance, the band 3 dimer can attach to the cytoskeleton via a complex of adducins, protein 4.1, and actin and form the junctional complex [9, 14]. After confirming that tyrosine at amino acid position 21 of band 3 was highly phosphorylated under physiological conditions in the HAPC group by a specific phospho-band 3 (Tyr21) antibody [13], we hypothesized that the tyrosine phosphorylation of band 3 would worsen the storage quality of SRBCs by activating glycolysis (and thus inhibiting the pentose phosphate pathway) and affecting the mechanical properties of RBCs in the Tibetan HAPC population.

## Materials and methods

### Study design

The research protocol was approved by the ethics committee of the Hospital of Chengdu Office of the People’s Government of Tibetan Autonomous Region (registration number: 2017-34) and the Institute of Blood Transfusion ethics committee (registration number: 201712). Written informed consent was obtained from all volunteers who participated in this study according to the Declaration of Helsinki. Volunteers were recruited from the Hospital of Chengdu Office of the People’s Government of Tibetan Autonomous Region, People’s Hospital of Tibet Autonomous Region and Chengdu Blood Center and divided into the HAC, HAPC and LLC groups according to hemoglobin concentration and altitude of residence. The inclusion criteria for the three groups are shown in Table 1. Tibetan volunteers were transferred to Chengdu, and blood was collected within 30 days of leaving the Tibetan Plateau. Individuals with a prior history of thrombus or hemorrhage, the use of oral anticoagulant therapy, hepatic disease, HIV/HBV/HCV/TP infection, pregnancy, diabetes or other organic diseases were excluded from this study. The sex, age and whole-blood count data from the three groups of volunteers are shown in Table 2.

**Table 1:** The inclusion criteria for the HAC, HAPC and LLC groups

	HAC group	HAPC group	LLC group
Age limit	18–55 years of age	18–55 years of age	18–55 years of age
Residence	resident of Tibet (> 3600 m) for more than 10 years	Resident of Tibet (> 3600 m) for more than 10 years	Resident of Chengdu (≈ 500 m)
Hemoglobin concentration requirement	120 g/L < males ≤ 185 g/L, 115 g/L < females ≤ 165 g/L	Males ≥ 210 g/L, Females ≥ 190 g/L	120 g/L < males ≤ 185 g/L, 115 g/L < females ≤ 165 g/L

**Table 2:** Sex, age and whole-blood count data for the HAC, HAPC and LLC groups

	HAC group	HAPC group	LLC group
Samples, (*n*)	18	12	25
Sex	15 male, 3 female	12 male	12 male, 13 female
Age	41.1 ± 8.2	44.4 ± 7.9	40.1 ± 11.1
WBC (× 10^9^/L)	6.04 ± 1.42	6.77 ± 4.14	5.73 ± 1.29
RBC (× 10^12^/L)	5.00 ± 0.48	6.76 ± 1.00	4.65 ± 0.56
PLT (× 10^9^/L)	226 ± 46	122 ± 36	176 ± 49
HGB (g/L)	157 ± 13	228 ± 16	143 ± 17
HCT (%)	46.6 ± 3.5	63.3 ± 6.8	43.1 ± 4.4
MCV	93.3 ± 3.7	94.4 ± 7.3	92.7 ± 2.4
MCH (pg)	31.5 ± 1.1	32.1 ± 3.7	31.0 ± 1.1
MCHC (g/L)	338 ± 6	339 ± 17	332 ± 10
RDW-SD (fL)	42.8 ± 1.9	52.6 ± 8.4	43.1 ± 2.1
RDW-CV (%)	12.8 ± 0.8	15.8 ± 3.8	13.1 ± 0.6

*WBC* white blood cell count, *RBC* red blood cell count, *PLT* platelet count, *HGB* hemoglobin, *HCT* hematocrit, *MCV* mean corpuscular volume, *MCH* mean corpuscular hemoglobin, *MCHC* mean corpuscular hemoglobin concentration, *RDW-SD* red blood cell distribution width-standard deviation value, *RDW-CV* red blood cell distribution width-coefficient of variation value

### Whole-blood collection, SRBC processing and storage

Whole blood (200 mL ± 10%) was collected into blood collection packs containing citrate–phosphate-dextrose-adenine-1 (CPDA-1) anticoagulant (28 mL) (Nan Geer Biomedical Corporation, Sichuan, China) according to standard procedures. Then, 10 mL of whole blood was drawn from each bag, and washed RBC samples were prepared from the whole blood by leucocyte filtration and saline washing. RBC ghost protein preparation and ECAR assay were performed on the day of blood collection using washed RBC samples.

The remaining whole blood was centrifuged at 3500 × g for 10 min at 4 °C. The plasma was removed, and 50 mL of mannitol-adenine-phosphate (MAP) additive solution was added to the packed RBCs to obtain the SRBCs. The three groups of SRBC units were stored under standard blood bank conditions at 4 °C in Chengdu and analyzed aseptically on days 1, 14, 21, and 35 of storage.

### Routine RBC quality assessment

Blood routine tests were performed on an automated hematology analyzer (BC-5800; Mindray, Shenzhen, China).

Supernatant potassium (K^+^), sodium (Na^+^), glucose and lactate concentrations were measured on an automatic biochemical analyzer (7180; Hitachi Co., Ltd, Japan) according to the manufacturer's instructions. Extracellular pH was measured with a pH meter (FE20; Mettler Toledo, Switzerland).

### ECAR assay

The ECAR was measured in a Seahorse XFe24 analyzer as previously reported with slight modifications [15]. For RBC samples, 5 × 10^6^ cells per well were plated onto XF24 cell plates coated with Cell-Tak (354242, Corning, New York, USA) in XF RPMI assay medium pH 7.4 (100 μL, XF assay medium) supplemented with glucose (40 mM), sodium pyruvate (1 mM) and L-glutamine (2 mM) and left for 10 min at room temperature. After seeding, the cell plates were centrifuged for 1.5 min at 200 × g at room temperature with an acceleration of one without braking. Afterward, 400 μL of XF assay medium was added to the cells, and the cells were incubated for 30 min at 37 °C without CO_2_. XF measurements included serial injections of glucose (10 mM), oligomycin A (5 μM) and 2-deoxy-glucose (2-DG, 50 mM).

### Pyruvate kinase (PK) activity assay

PK activity was tested using a commercial PK assay kit according to the manufacturer’s instructions (ab83432, Abcam).

### Preparation of RBC ghost proteins

RBC ghost proteins were prepared as previously described [16]. In brief, packed RBCs were lysed in 0.01 mol/L hypotonic Tris–HCl solution for 2 h. Then, the lysates were centrifuged at 10,000 × g for 30 min. After discarding the supernatant, the sediment containing RBC ghost proteins was resuspended in lysis solution, and this step was repeated three times. The concentration of RBC ghost proteins was determined with a Bradford protein assay kit (P0010, Beyotime, China).

### SDS‒PAGE and western blotting

Aliquots of RBC ghost proteins were separated by 10% SDS‒PAGE, with each lane containing 8 μg of protein, and electrophoretically transferred to a polyvinylidene difluoride membrane (Millipore Immobilon-PSQ). Blocking was performed for 4 h at room temperature in 5% (w/v) bovine serum albumin. Incubation with monoclonal phospho-band 3 (Tyr21) antibody (ab125070, dilution 1: 2000; Abcam) was performed overnight at 4 °C in 2.5% (w/v) bovine serum albumin in TBS (containing 0.1% Tween-20). Bands were detected with HRP-linked anti-rabbit IgG antibody (#7074; dilution 1:2000; CST) using a Super Signal West Pico chemiluminescence detection kit (Thermo Scientific) and digitized with ImageQuant LAS 4000mini (GE Healthcare).

### Hemolysis assay

Briefly, the free hemoglobin concentration in the supernatant was assessed by the orthotolidine method using a spectrophotometer (Ultrospec 6300 Pro; Amersham Biosciences, United States). The hemolysis rate is expressed as a percentage of the total Hb present in RBCs after correcting for the Hct. The hemolytic rate (%) was calculated using the following standard formula: hemolytic rate (%) = (100—HCT) × Hb _supernatant_/Hb _total_ [17].

### 2,3-DPG assay

A 2,3-diphosphoglycerate (2,3-DPG) assay was carried out using commercial 2,3-DPG kits according to the manufacturer's instructions (Roche Diagnostics, Mannheim, Germany).

### RBC deformability measurements

RBC deformability was measured using a laser-assisted optical rotational cell analyzer (Lorrca; Maxsis, The Netherlands) as previously described [18, 19]. In brief, RBCs were diluted 1:100 in a polyvinylpyrrolidone solution and subjected to increasing shear stress (0.30–30.00 Pa) at 37.0–37.5 °C. The elongation index (EI) was collected at each stress level.

### Scanning electron microscopy (SEM)

SEM studies of RBC morphology were performed using a Quanta 250 electron microscope (FEI Company). RBC samples (randomly chosen from each group) were fixed in phosphate-buffered (pH 7.4) 2.5% glutaraldehyde for 2 h, mounted on mica slides, washed twice in 0.1 M phosphate buffer (pH 7.4), and then dehydrated in graded ethanol (25, 50, 70, 80, 90, and 100%). After natural drying and covering with a gold–palladium layer, the samples underwent SEM analysis.

### Statistical analysis

The results are shown as the mean ± standard deviation (SD). Statistical analysis was performed using SPSS statistics software, version 21.0. ANOVA was used to compare the blood biochemical indexes of the three groups of SRBCs stored for the same duration, and Bonferroni correction for multiple comparisons was conducted. A p value < 0.05 was considered to indicate statistical significance.

## Results

### Parameters of the three groups of SRBCs

**Fig. 1:**
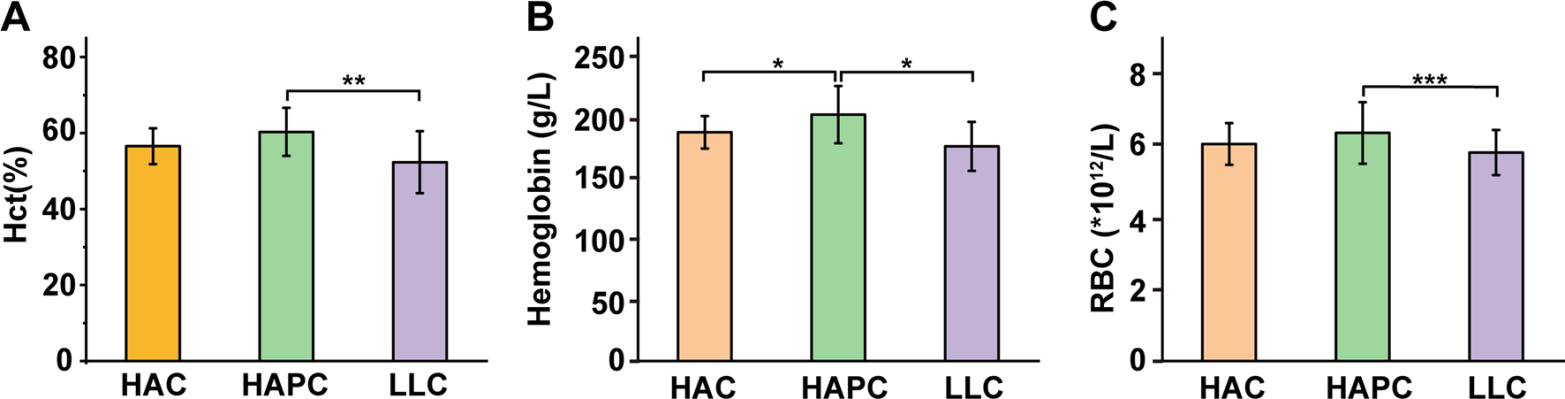
Hematocrit (**A**), HGB concentration (**B**), and RBC count (**C**) of SRBCs in the three groups. The HAC and HAPC groups correspond to the Tibetan population, and LLC group D corresponds to the Han population dwelling in Chengdu. Asterisks indicate statistical significance (ANOVA with Bonferroni correction for multiple comparisons—* p < 0.05; ** p < 0.01; *** p < 0.001)

As shown in Fig. 1, because the whole blood collected from the Tibetan HAPC population had a remarkably high hemoglobin concentration, the Hct (Fig. 1A), HGB (Fig. 1B), and RBC count (Fig. 1C) of SRBCs prepared from the HAPC group were significantly higher than those prepared from the LLC group (p < 0.05), and the total HGB of SRBCs prepared from the HAPC group was also higher than that of the HAC group (p < 0.05). Meanwhile, there was no significant difference in the erythrocyte-related parameters between the HAC and LLC groups.

### The glycolytic capacity of RBCs was increased in the HAPC group

**Fig. 2:**
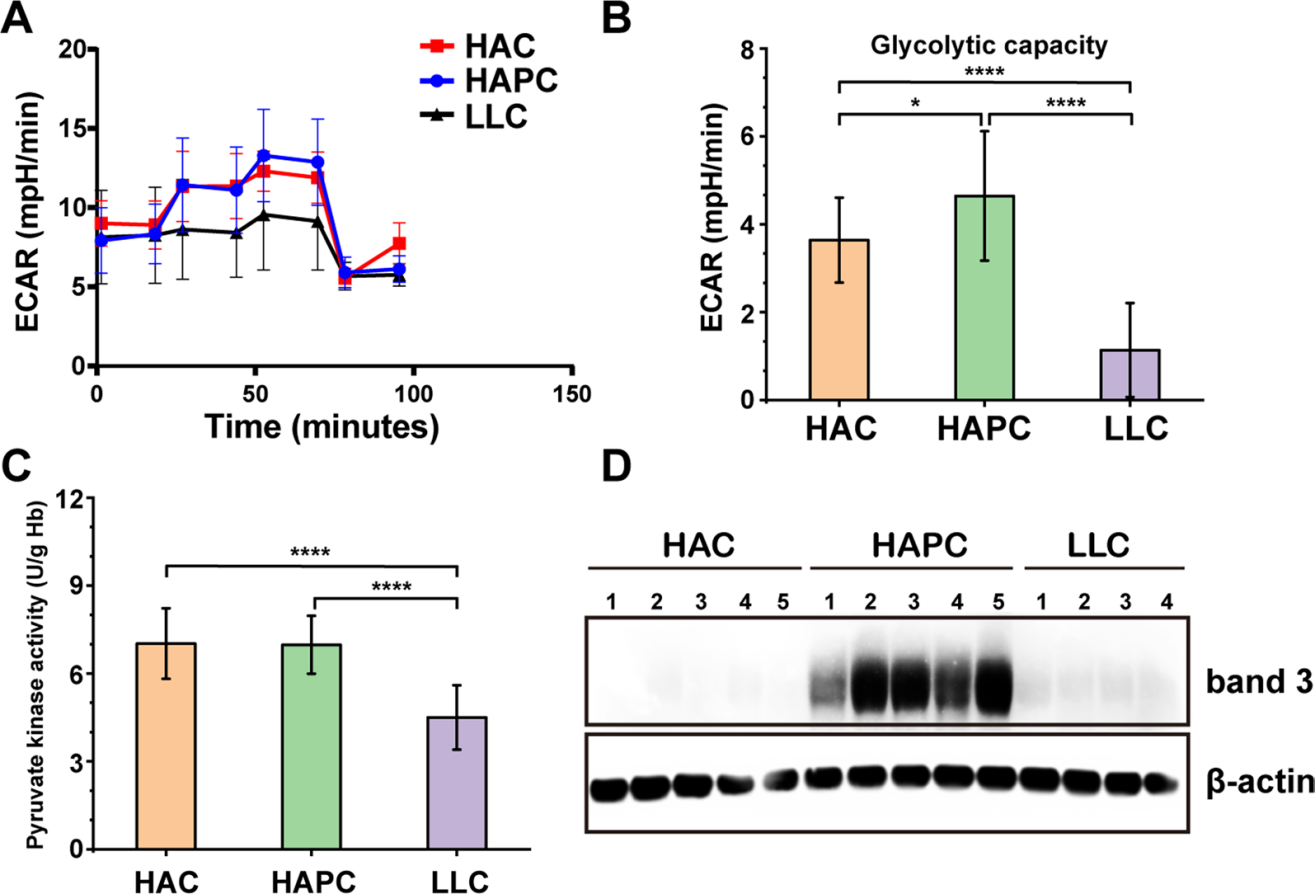
ECAR assay (**A**), glycolytic capacity (**B**), and PK activity (**C**), and tyrosine phosphorylation of band 3 (**D**) of RBCs in the HAC, HAPC and LLC groups. Asterisks indicate significance (ANOVA with Bonferroni correction for multiple comparisons—* p < 0.05; **** p < 0.0001)

We analyzed the extracellular acidification rate (ECAR) of the three groups of RBCs on the day of blood collection (Fig. 2A), and the glycolytic capacity of the three groups of RBCs was calculated (Fig. 2B). The glycolytic capacity of RBCs in the HAPC group was the highest among the three groups, while the glycolytic capacity of RBCs in the LLC group was the lowest. To investigate the mechanism underlying the increased glycolytic capacity of RBCs in the HAPC group, we further analyzed the PK activity of the three groups of RBCs. As PK is a key rate-limiting glycolytic enzyme, the glycolytic capacity was significantly affected by PK activity, which was detected by a commercial assay kit according to the manufacturer's instructions. There was no significant difference in PK activity between the HAPC and HAC groups; however, both groups showed higher PK activity than the LLC group (Fig. 2C).

### Tyrosine 21 of band 3 was highly phosphorylated under physiological conditions in the HAPC group

As noted above, RBC ghost proteins were prepared on the day of blood collection. With a specific phospho-band 3 (Tyr21) antibody (ab125070, Abcam, United Kingdom), we analyzed the tyrosine phosphorylation of band 3 on the RBC membrane in the three groups by western blotting. In addition, more apparent phosphorylation bands in the region of 100  kDa were observed for the HAPC group (Fig. 2D). Thus, Y21, which is located in the cytoplasmic domain of band 3, was highly phosphorylated in the HAPC group under physiological conditions.

### Changes in SRBC storage quality among the three groups under standard blood bank conditions

**Fig. 3:**
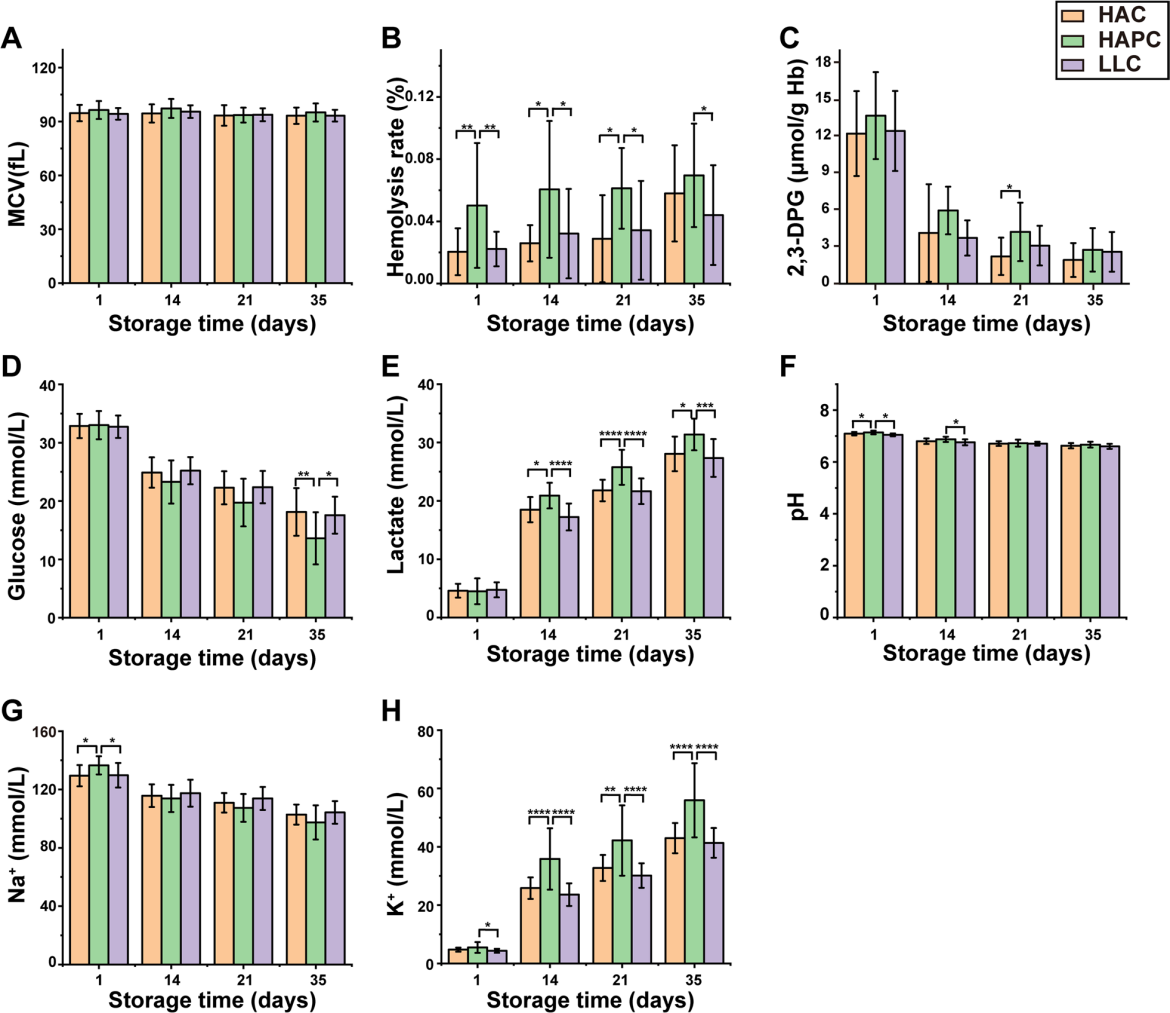
Storage quality parameters of the three groups of SRBCs. The mean corpuscular volume (**A**), hemolysis rate (**B**), 2,3-diphosphoglycerate level (**C**), glucose level (**D**), lactate level (**E**), pH (**F**), and supernatant sodium and potassium levels (**G**, **H**) were measured throughout the 35 day storage period. Asterisks indicate significance (ANOVA with Bonferroni correction for multiple comparisons—* p < 0.05; ** p < 0.01; *** p < 0.001; **** p < 0.0001)

The storage quality of the three groups of SRBCs was analyzed aseptically on the 1st, 14th, 21st, and 35th days of storage. There was no significant difference in mean corpuscular volume (MCV) among these three groups of SRBCs at any time point (Fig. 3A). The hemolysis rate of the three groups of SRBCs increased with prolonged storage time, and the hemolysis rate of the HAPC group was significantly higher than that of the HAC and LLC groups throughout the storage time (p < 0.05) (Fig. 3B). The 2,3-DPG levels in the three groups of SRBCs decreased with prolonged storage time. The 2,3-DPG levels of the HAPC group were higher than those of the HAC group on the 21st day of storage (p < 0.05), whereas no significant difference was found among the three groups at other time points (Fig. 3C). Glucose is the energy source for RBCs, but there was no significant difference in the supernatant glucose concentration among the three groups of SRBCs on the 1st day of storage, and the glucose concentration decreased with prolonged storage time (Fig. 3D). Moreover, the glucose concentration of the HAPC group was significantly lower than that of the HAC and LLC groups on the 35th day of storage (p < 0.05). This meant that RBCs of the HAPC group consumed more glucose during storage. Additionally, the end product of glycolysis in RBCs, lactate, gradually accumulated in the supernatant during storage. In the middle and terminal stages of storage (the 14th, 21st and 35th days), the lactate concentration of the HAPC group was significantly higher than that of the HAC and LLC groups (p < 0.05) (Fig. 3E). The pH of the three groups of SRBCs decreased with prolonged storage time. Moreover, the pH of the HAPC group was significantly higher than that of the HAC and LLC groups on the 1st day of storage, and the pH of the HAPC group was higher than that of the LLC group on the 14th day of storage (p < 0.05). However, no significant difference in pH was found among the three groups in the middle or terminal stages of storage (21st and 35th days, respectively), which may be because the additive solution applied to the SRBCs has a strong buffering capacity (Fig. 3F). SRBCs of the HAPC group had higher Na^+^ levels in the supernatant than those of the HAC and LLC groups on day 1 (p < 0.05); however, there was no significant difference in Na^+^ levels among these three groups at the middle or terminal stage of storage, probably due to poor activity of the Na^+^-K^+^ ATP enzyme in erythrocytes in the HAPC group. On the other hand, SRBCs of the HAPC group had the highest supernatant K^+^ levels among those of the three groups in the middle and terminal stages of storage (p < 0.01), probably because the HAPC group had the highest hemolysis rate, as hemolysis releases K^+^ into the supernatant (Fig. 3G, H).

**Fig. 4:**
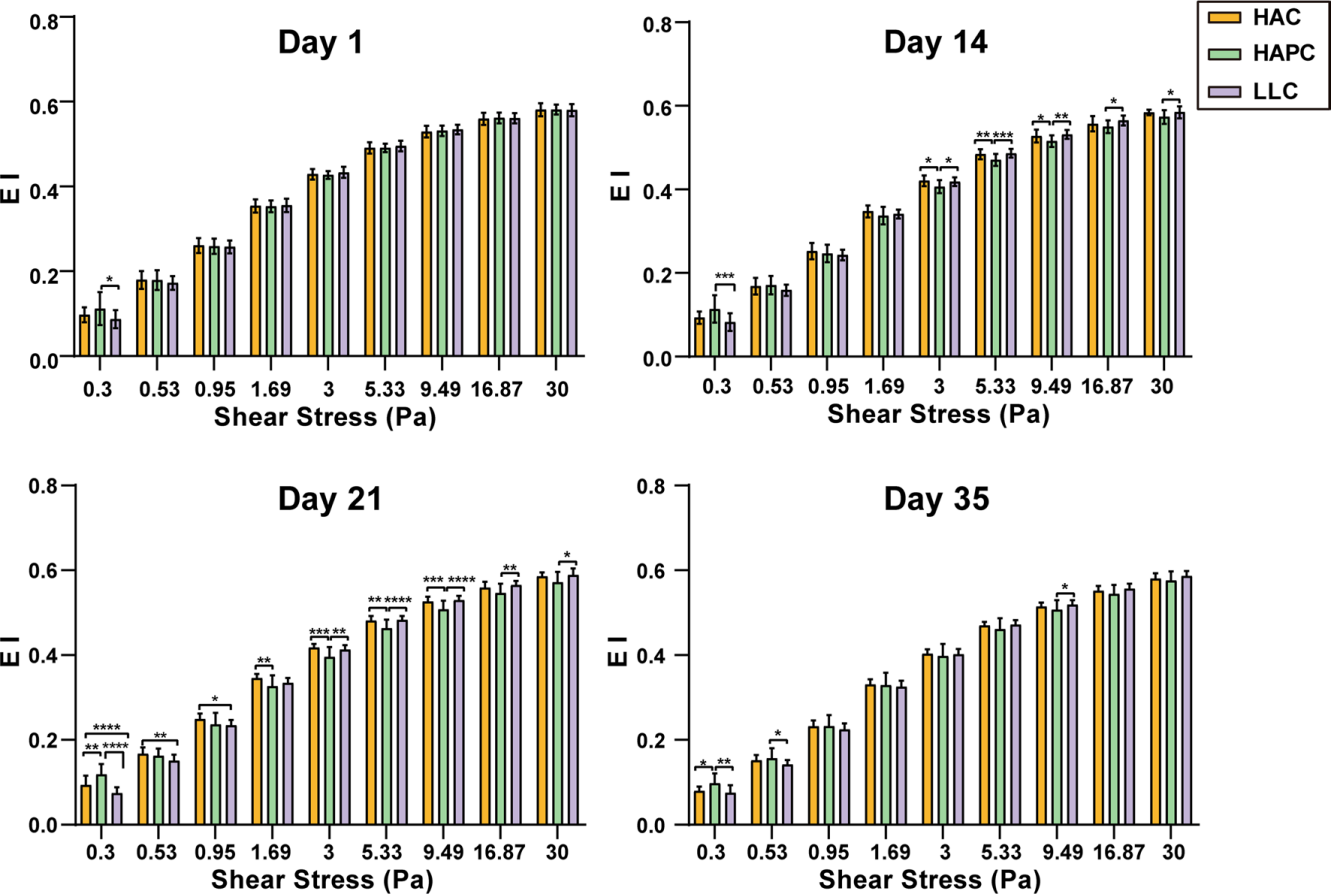
Deformability of the three groups of SRBCs under different shear stresses at each time point. Asterisks indicate significance (ANOVA with Bonferroni correction for multiple comparisons—* p < 0.05; ** p < 0.01; *** p < 0.001; **** p < 0.0001)

The RBC deformability curves for the three groups of SRBCs over the course of storage were also analyzed. With prolonged storage time, the RBC deformability of the three groups of SRBCs showed similarly decreasing EI values under different shear stresses ranging from 0.3 to 30 Pa. Surprisingly, the RBC deformability of the HAPC group was higher than that of the LLC group under low shear stress (0.3 Pa) throughout the storage time (p < 0.05), while the RBC deformability of the HAPC group was lower than that of the HAC and LLC groups under high shear stress (3, 5.33, 9.49, 16.87 and 30 Pa) at days 14 and 21 of storage and 9.49 Pa at day 35. Moreover, a significant difference in RBC deformability between the HAC and LLC groups was found only at low shear stress (0.3, 0.53 and 0.95 Pa) at day 21 (Fig. 4).

**Fig. 5:**
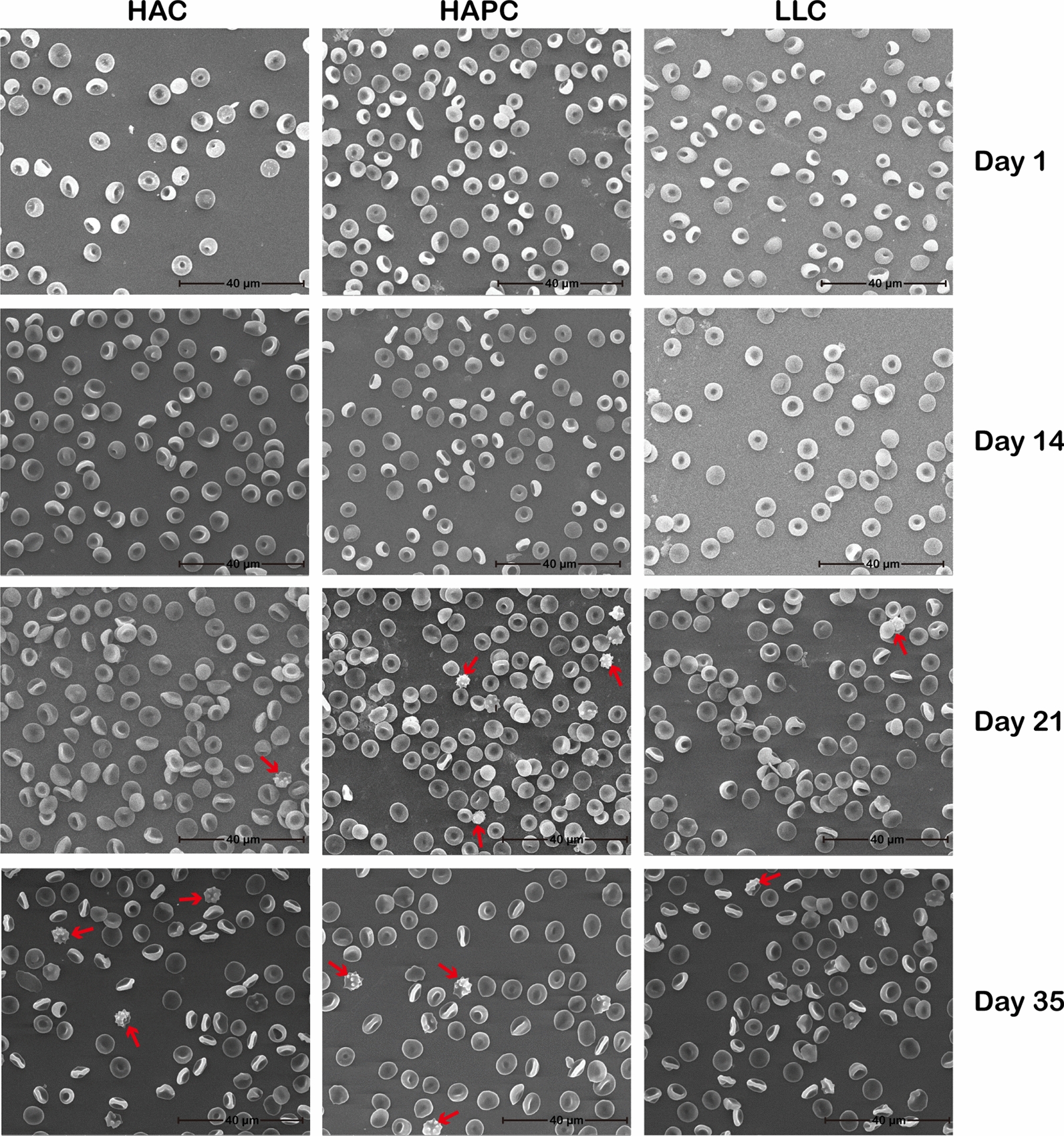
SEM of three stored groups of SRBCs at each time point. Acanthocytes are indicated by red arrows. Magnification: × 3000. Scale bar: 40 μm

The RBC morphology of the three groups of SRBCs at each time point of storage was detected by scanning electron microscopy. There was no significant difference in RBC morphology between the three groups of SRBCs in the early stage of storage, and all of the erythrocytes were double concave disc-shaped. With prolonged storage time, the RBC morphology of the three groups of SRBCs varied in a similar way, showing an increasing proportion of acanthocytes (Fig. 5).

As noted before, the RBC number of SRBCs in the HAPC group was significantly higher than that of SRBCs in the HAC and LLC groups, and the metabolic parameters of the SRBCs, such as glucose, lactate, Na^+^ and K^+^ levels, can be influenced by RBC number. To exclude the influence of RBC number on these parameters, we proposed the following formula to calculate the rate of change in metabolic parameters during storage:

$${\varvec{rate \;of \; change \; inmetabolic \; parameters}}=\frac{|{\varvec{V}}{\varvec{day1}}-{\varvec{V}}{\varvec{day35}}|}{{\varvec{RBC}}\;{\varvec{Number}}}$$

where V_day1_ represents the value of the metabolic parameter on day 1 of storage, and V_day35_ represents the value of the metabolic parameter on day 35 of storage.

**Fig. 6:**
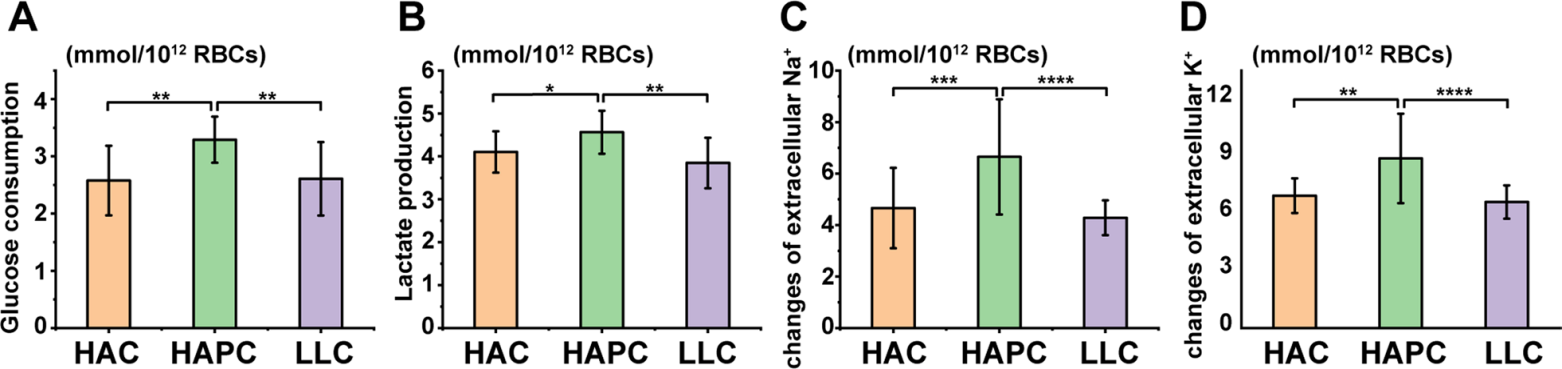
Rates of change in glucose (**A**), lactate (**B**), Na^+^ (**C**) and K^+^ (**D**) levels in the three groups of SRBCs throughout the storage period. Asterisks indicate significance (ANOVA with Bonferroni correction for multiple comparisons—** p < 0.01; *** p < 0.001; **** p < 0.0001)

By homogenizing the RBCs, the change rates of SRBCs metabolic parameters, including glucose (Fig. 6A), lactate (Fig. 6B), Na^+^ and K^+^ levels (Fig. 6C, D), were significantly different among the HAC, HAPC and LLC groups over the entire storage period. The SRBCs of the HAPC group clearly consumed more glucose and generated more lactate than those of the HAC and LLC groups, not because of higher hematocrit and RBC numbers but because of the metabolic characteristics of RBCs from the Tibetan HAPC population. Additionally, the enhanced RBC glycolysis in the HAPC group was strongly associated with the tyrosine phosphorylation of band 3.

## Discussion

The hemoglobin concentration of plateau residents often exhibits a compensatory increase to maintain the body's oxygen supply. However, increases in hemoglobin concentration and hematocrit pose a serious challenge to the storage quality of SRBCs prepared from the blood of the high-hemoglobin population in Tibet. To establish the quality criteria of SRBCs applicable to the Tibetan Plateau, a new in vitro storage study was carried out by our research group with SRBCs prepared from plateau residents with different hemoglobin concentrations.

In our previous small-scale blood storage experiment, we found significant differences in metabolic parameters between SRBCs prepared from the HAPC population and lowland population [8], which was consistent with the present study. In addition, several storage quality parameters, such as the hemolysis rate, change rates of Na^+^ and K^+^, showed similar variation tendencies in the two studies. In particular, SRBCs prepared from the blood of the Tibetan HAPC population generated more lactate during storage, which was an attractive phenomenon that had not been reported before. However, due to the different preparation times and groups of SRBCs, some different results of these two studies are shown in Tables 3, 4.

**Table 3:** Different collection and preparation conditions between the two studies

	Pervious 2015 study [8]	This study
Collection locality	Lhasa	Chengdu
Preparation time of SRBCs	One week after whole-blood collection	Within 6 h of whole-blood collection
Population	Han Chinese who migrated to the Tibetan plateau for more than 1 year	Local Tibetan volunteers, resident of Tibet (> 3600 m) for more than 10 years, and blood was collected within 30 days of leaving the Tibetan Plateau
Grouping	No further grouping was performed in high altitude population	Volunteers were divided into the high-altitude control (HAC) group (120 g/L < males ≤ 185 g/L, 115 g/L < females ≤ 165 g/L) and the HAPC group (males ≥ 210 g/L, females ≥ 190 g/L)

**Table 4:** Different results between the 2015 and present study

Storage quality parameters	Pervious 2015 study [8]	This study	Reason for difference
pH	From 1st day to 35th day, HAPC < LLC	On 1st day and 14th day, HAPC > LLC	The preparation time of HAPC SRBCs in 2015 was later than this study for one week
2,3-DPG	On 1st day and 7th day, HAPC > LLC, no results on 21st day and 35th day	On 1st, 14th, 21st day, HAPC > LLC	Different calculation method and different test time
Morphology of RBCs	the number of normally shaped discocytes in plateau SRBCs was much less than lowland SRBCs	the RBC morphology of the three groups of SRBCs varied in a similar way, showing an increasing proportion of acanthocytes	Different preparation time and groups of high altitude SRBCs

As mentioned above, in our 2015 study, whole-blood samples of the plateau group (HAPC group) had been stored at 4 ℃ for an extra week in Lhasa before being transported to Chengdu for subsequent preparation of SRBCs. This would lead to lactate accumulation in whole-blood samples of the HAPC group. After preparation into SRBCs, these SRBCs samples of the HAPC group would contain more lactate at the initial phase of storage. And the transportation process (such as vibration) from Lhasa to Chengdu may also have an impact on the quality of whole-blood samples. The above mentioned factors would impair the storage quality of SRBCs prepared from the HAPC group, resulting in differences in the variation tendencies of pH, 2,3-DPG and RBCs morphology between the two studies.

Therefore, in the present work, to exclude the influence of inconsistent preparation times of SRBCs and transportation processes on storage quality, blood from Tibetan volunteers was collected within 30 days of leaving the Tibetan Plateau for subsequent preparation of SRBCs. Second, Tibetan blood donors were divided into a high-altitude control (HAC) group and a HAPC group according to hemoglobin concentration. Moreover, we analyzed differences in glycolytic capacity among the HAC, HAPC and LLC groups and influencing factors that may affect glycolysis in RBCs, such as the activity of glycolytic enzymes and phosphorylation of band 3.

According to the results of the ECAR assay and changes in the storage quality of the SRBCs, we found that erythrocyte glycolysis in the HAPC group was significantly enhanced, which generated more lactate. In addition, with a specific phospho-band 3 (Tyr21) antibody, we found that tyrosine 21 of band 3 was highly phosphorylated under physiological conditions in the HAPC group.

Considering that band 3 tyrosine phosphorylation plays an important role in regulating the metabolic flux of erythrocytes [11–13], we propose that the phosphorylation of Y21 under physiological conditions in the Tibetan HAPC population releases glycolytic enzymes from the membrane to the cytoplasm, thus activating glycolysis and generating more lactate during storage. Although the sample size of this study was not large enough, tyrosine phosphorylation of Y21 was found in all HAPC samples, suggesting that activation of glycolysis regulated by tyrosine phosphorylation of band 3 was prevalent in the Tibetan HAPC population.

The tyrosine phosphorylation level of band 3 is regulated by the actions of members of two opposing enzyme families, phosphotyrosine kinases (PTKs) and phosphotyrosine phosphatases (PTPs), at specific tyrosine sites. In general, PTP activity is much higher than PTK activity, so a very low basal level of tyrosine phosphorylation is maintained in RBCs. The tyrosine kinases Lyn and Syk interact with the cytoplasmic domain of band 3, which leads to tyrosine phosphorylation [20]. The phosphorylation of band 3 primarily occurs at Y8 and Y21, while secondary phosphorylation affects Y359 and Y904 [13]. In addition, the phosphorylation of Y21 in the HAPC group indicates the disruption of homeostatic balance between PTKs and PTPs.

Two lines of evidence demonstrate that the phosphorylation of Y21 can affect the storage quality of SRBCs. First, ECAR data showed that the glycolytic capacity of RBCs was higher in the HAPC group among the three groups of volunteers, while there was no significant difference in PK activity between the HAPC and HAC groups. Campanella reported that the cytosolic domain of band 3 contains binding sites for glycolytic enzymes and deoxyhemoglobin, and the phosphorylation of Y21 reversibly releases all glycolytic enzymes (including glyceraldehyde-3-phosphate dehydrogenase, aldolase, and phosphofructokinase) from the membrane [21]. Glycolytic enzymes translocate from the RBC membrane to the cytoplasm and activate glycolysis to generate more lactate; simultaneously, the pentose phosphate pathway is inhibited in RBCs. Apart from this, SRBCs of the HAPC group consumed more glucose and generated more lactate than those of the HAC and LLC groups during storage, which is evidence of glycolytic activation. Therefore, we believe that glycolytic activation and lactate production mediated by the tyrosine phosphorylation of band 3 resulted in a reduction in storage quality in SRBCs from the HAPC group. Second, the RBC deformability of the HAPC group decreased more significantly under high shear stress than that of the HAC and LLC groups, and this was accompanied by the highest hemolysis rate in the HAPC group during storage. Ferru demonstrated that the tyrosine phosphorylation (especially Y8 and Y21) of band 3 triggers its dissociation from ankyrin and consequent release from the spectrin/actin skeleton, thus affecting the mechanical properties of RBCs. The weaker RBC deformability of the HAPC group under high shear stress supports the above theory [18, 20, 22].

In previous reports, researchers have found that sickle cell disease, thalassemias and glucose-6-phosphate dehydrogenase deficiency lead to enhanced tyrosine phosphorylation of band 3 [23–25]. However, the tyrosine phosphorylation of band 3 under physiological conditions in the Tibetan HAPC population has been reported here for the first time. It is important to distinguish the tyrosine phosphorylation (Y21) of band 3 under physiological conditions in the Tibetan HAPC population from the aggregation and Ser/Thr phosphorylation of band 3 caused by storage lesions [26].

In summary, the glycolytic activation of RBCs resulting from band 3 tyrosine phosphorylation is a metabolic characteristic of the Tibetan HAPC population. Previous studies have shown that in the process of acclimatization, erythrocyte sphingosine kinase 1 (Sphk1) induces the accumulation of sphingosine-1-phosphate in RBCs, which prompts the binding of deoxyhemoglobin to the RBC membrane, thus promoting the release of glycolytic enzymes into the cytoplasm and enhancing glycolysis [27]. However, our study showed that band 3 tyrosine phosphorylation could also release glycolytic enzymes from the membrane to the cytoplasm, thus facilitating glycolysis in the Tibetan HAPC population. These studies have shown that RBC metabolism reprogramming plays an important role in altitude acclimatization and is regulated by multiple mechanisms. Additionally, band 3 tyrosine phosphorylation impairs the storage quality of SRBCs by activating glycolysis and decreasing RBC deformability in the Tibetan HAPC population. Elucidating the regulatory mechanism of the tyrosine phosphorylation and dephosphorylation of band 3 in the Tibetan HAPC population is also critical for revealing new therapeutic targets for HAPC treatment. However, band 3 tyrosine phosphorylation poses a serious challenge to the storage quality of SRBCs prepared from the blood of the high-hemoglobin population in Tibet. We will try to optimize SRBC storage technology according to the characteristics of erythrocyte metabolism in the high-hemoglobin Tibetan population in the future.

## Data Availability

The data that support the findings of this study are available on request from the corresponding author.
